# A point-prevalence surveillance of healthcare-associated infections in a tertiary care teaching hospital in Malaysia

**DOI:** 10.1017/ash.2025.121

**Published:** 2025-09-03

**Authors:** Fauziah Ismail, Nor Reah Mustafa, Maimunah Hasan, Fauzi Idris, Noraida Muhammad, Azrahamiza Abdullah, Sharizan Awang, Nurul Iza Mat Zan, Hakimah Ab Kadir, Wan Noor Faradilla Mat Latif, Che Siti Noraini Abdullah, Yuslizawati Mohd Shaufi, Syaidatull Asmara Aminudden, Silamai A/P Ea Chum, Siti Suraiya

**Affiliations:** 1 Infection Control Unit, Hospital USM Universiti Sains Malaysia; 2 Department of Medical Microbiology and Parasitology, School of Medical Sciences Universiti Sains Malaysia

## Abstract

**Background:** Prevention of Healthcare-Associated Infections (HCAIs) is an essential component of patient’s safety in every healthcare setting and serve as an indicator for a good healthcare practice. Surveillance for HCAIs is important to measure their burden, identify high-risk patients and procedures, and guide efforts to reduce HCAI incidence. The aim of current study is to determine the prevalence of HCAI Hospital Universiti Sains Malaysia (Hospital USM), Kelantan, Malaysia. **Methods:** A one-day point prevalence survey (PPS) was conducted between 1st October 2023 to 15th October 2023 on all patients admitted to 15 selected wards at Hospital USM. The PPS was performed strictly following the “Manual for point prevalence survey for healthcare associated infection” by Ministry of Health Malaysia. Data were collected by a team of trained infection control practitioners, compiled, and analysed accordingly. **Results:** The surveyed hospital is a tertiary care teaching hospital contained 829 beds, has 11 certified infection control nurses, has 50 isolation rooms and 4 negative pressure rooms. During the surveyed period, there were 121 patients on continuous bladder catheterization, 63 patients had central venous catheter in situ and 107 patients were on mechanical ventilation.

Of the 588 patients surveyed, 14 (2.4%) had an active HCAI. Identified predisposing factors associated with the occurrence of HCAI were underlying medical illness (40.7%), prolonged hospitalization (25.9%), prematurity (11.1%), history of surgery (11.7%), immunosuppressive therapy (7.4%) and others (3.7%). The most frequent types of HCAI were pneumonia, followed by blood stream infection, clinical sepsis, surgical site infection and urinary tract infection. **Conclusions:** The survey reports an overall prevalence of 2.4% of HCAI in Hospital USM. A yearly PPS is very useful tool to measure the overall prevalence of HCAI, highlighting the areas with prevalence that require special attention and allowing planning for improvement actions.

